# Molecular epidemiology and clinical profiles of carbapenem-resistant Enterobacterales in neonates from two large children’s hospitals in southwestern China

**DOI:** 10.3389/fcimb.2026.1735917

**Published:** 2026-05-21

**Authors:** Jia Zhang, Haiping Wang, Huimin Chang, Hanyi Wang, Yinli Zhuo, Xiuyu Xu, Xuemei Zhang, Qun Zhang

**Affiliations:** 1 Department of Clinical Laboratory, Children’s Hospital of Chongqing Medical University, National Clinical Research Center for Children and Adolescents' Health and Diseases, Ministry of Education Key Laboratory of Child Development and Disorders, Chongqing Key Laboratory of Pediatric Metabolism and Inflammatory Diseases, Chongqing, China; 2 Department of Laboratory Medicine, Key Laboratory of Diagnostic Medicine (Ministry of Education), Chongqing Medical University, Chongqing, China; 3 Department of Clinical Laboratory, Kunming Children’s Hospital, Kunming, China; 4 Basic Medicine Research and Innovation Center for Novel Target and Therapeutic Intervention, Ministry of Education, College of Pharmacy, Chongqing Medical University, Chongqing, China; 5 Department of Laboratory Medicine, The First Affiliated Hospital of Chongqing Medical University, Chongqing, China

**Keywords:** antimicrobial resistance, carbapenem-resistant Enterobacterales, molecular epidemiology, neonate, risk factors

## Abstract

**Introduction:**

Carbapenem-resistant Enterobacterales (CRE) are a global health threat due to their high antimicrobial resistance and transmissibility. However, data on CRE infection in neonates in China are lacking. We collected non-duplicate CRE isolates from neonates between 2018 and 2022 in Chongqing and Kunming and investigated their antimicrobial resistance profiles, risk factors, and molecular epidemiology.

**Methods:**

Antimicrobial susceptibility testing was performed using broth microdilution. Polymerase chain reaction (PCR) was performed to detect carbapenem-resistance genes, extended-spectrum β-lactamase (ESBL) genes, and virulence genes. Multilocus sequence typing (MLST) was used to analyze strain relatedness. Clinical data were collected and multivariable logistic regression analysis was used to identify risk factors.

**Results:**

A total of 139 non-duplicate neonatal CRE isolates were collected from 2018 to 2022, comprising carbapenem-resistant *Klebsiella pneumoniae* (CRKP, 81.3%) and carbapenem-resistant *Escherichia coli* (CREC, 18.7%). New Delhi metallo-β-lactamase (NDM) was the predominant carbapenemase (83.5%) in neonates, followed by imipenemase (IMP, 11.5%) and *Klebsiella pneumoniae* carbapenemase (KPC, 4.3%). Extended-spectrum β-lactamase (ESBL) genes (including *bla_SHV_, bla_TEM,_* and *bla_CTX-M_*) were widely distributed among these isolates. Sequence type 2 (ST2, 84.6%) was the most prevalent among CREC isolates, while ST2407 (15.9%) was the dominant sequence type among CRKP isolates. Four novel STs were identified. The detection rate of hypervirulent *K. pneumoniae* (hvKP) was 8.0% in neonatal CRKP isolates. All isolates exhibited high-level resistance to cephalosporins and carbapenems. However, resistance rates to amikacin, tigecycline, colistin, and aztreonam-avibactam remained relatively low. Cesarean section (OR 3.283; 95% CI 1.682–6.411), elevated white blood cell (WBC) count (OR 1.049; 95% CI 1.001–1.100), and patent ductus arteriosus (OR 2.758; 95% CI 1.374–5.534) were significantly associated with an increased risk of neonatal CRE infection, whereas increasing age was associated with reduced risk thereof (OR 0.965; 95% CI 0.934–0.998); all *P* < 0.05).

**Conclusion:**

NDM is highly prevalent and transferable among CRE isolates in neonatal infections. The high-risk ST2407 NDM-producing *K. pneumoniae* clone warrants increased attention and monitoring of neonates. Aztreonam-avibactam (ATM/AVI) demonstrated potent *in vitro* activity against neonatal CRE isolates. Cesarean delivery, age, WBC count, and patent ductus arteriosus were identified as key risk factors for neonatal CRE infection.

## 1 Background

Carbapenem-resistant *Enterobacterales* (CRE) have emerged as a critical threat in hospital-acquired infections, particularly among vulnerable neonatal populations. These multidrug-resistant pathogens predominantly cause urinary tract, respiratory tract, bloodstream, and soft tissue infections. Mortality rates for neonatal sepsis caused by CRE have been reported to range from 29% to 43% (Ballot et al., 2019). The rapid global spread of CRE has been driven by the horizontal transfer of carbapenemase genes. Surveillance data from the China Antimicrobial Surveillance Network (CHINET) reveal a striking increase in imipenem resistance rates among *Klebsiella pneumoniae* isolates in China, rising from 3.0% in 2005 to 22.6% in 2024 (Qin et al., 2024). This alarming trend has prompted the World Health Organization to classify CRE as priority pathogens urgently requiring new therapeutics. ^1^,^2^.

The molecular epidemiology of CRE exhibits distinct patterns across different patient groups and regions. In China, sequence type 11 (ST11) *Klebsiella pneumoniae* producing *Klebsiella pneumoniae* carbapenemase-2 (KPC-2) is dominant in adults (Zhang et al., 2017). However, significant regional differences have been observed in broader pediatric populations. For example, it has been reported that in the eastern provinces of China, ST11 KPC-producing *Klebsiella pneumoniae* a high-risk clone among the children populations, while in the southwestern provinces of China, sequence type 789 (ST789) New Delhi metallo-β-lactamase (NDM)-producing *Klebsiella pneumoniae* is the predominant clone among the pediatric population (Wu et al., 2023). However, our understanding of the characteristics of neonatal CRE infection is still limited. Available single-center studies suggest that NDM-type carbapenemases may be more prevalent in neonates (Wu et al., 2023), but comprehensive multicenter data are lacking. This knowledge gap is particularly concerning given neonates’ unique vulnerabilities, including immature immune systems, limited antibiotic options due to toxicity concerns (e.g., avoidance of tigecycline and polymyxins), and heightened susceptibility to invasive device-related infections (Bassetti et al., 2020; Wang et al., 2022; Yin et al., 2023).

The clinical management of neonatal CRE infections faces three major challenges: (1) new β -lactam/β -lactamase inhibitor combinations (e.g., ceftazidime-avibactam, aztreonam-avibactam) lack clinical susceptibility data specific to neonates; (2) hypervirulent carbapenem-resistant *Klebsiella pneumoniae* strains (CR-hvKP), while rare in neonates, can cause devastating outcomes when they occur (Li et al., 2021); and (3) traditional infection control strategies derived from adult studies may not address neonatal-specific risk factors such as gestational age and mode of delivery.

In this study, we investigated the clinical characteristics and risk factors associated with neonatal CRE infection, analyzed the antimicrobial resistance profiles and epidemiological characteristics of CRE isolates, including carbapenem resistance genes, extended-spectrum β-lactamases genes, virulence genes, and multilocus sequence typing, to provide evidence-based insights for the prevention and control of neonatal CRE infection.

## 2 Materials and methods

### 2.1 Patients

This retrospective study collected basic clinical information on hospitalized neonates infected with CRE and carbapenem-susceptible *Enterobacteriaceae* (CSE) in the neonatal wards of the Children’s Hospital of Chongqing Medical University and Kunming Children’s Hospital between 2018 and 2022. The Children’s Hospital of Chongqing Medical University is one of the largest pediatric hospitals in China, where over 8000 neonates are hospitalized every year. Kunming Children’s Hospital is a children’s medical center in Yunnan Province, China, where over 7000 neonates are treated every year. We defined neonates as infants no older than 28 days. All included CRE group were from patients with documented clinical signs of infection (e.g., fever, respiratory symptoms, and abnormal laboratory findings), together with positive cultures from normally sterile sites or other clinically significant specimens. The CSE control group was selected from the same population of neonates diagnosed with Enterobacteriaceae infection during the study period and was frequency-matched to CRE cases by hospital and year of isolation. Inclusion criteria: (1) age between 0 days and 28 days; (2) diagnosis of Enterobacteriaceae infection, categorized as either CRE or CSE based on susceptibility testing results; (3) complete medical records available. Exclusion criteria: (1) patients not diagnosed with Enterobacteriaceae infection; (2) community-acquired colonization and infections from the outpatient department; (3) missing key data and subsequent episodes in the same patient; (4) CSE cases were excluded if they had any documented CRE-positive cultures during the study period, to ensure clear differentiation between the CRE and CSE groups. For inpatients with multiple episodes of infection with CRE or CSE, only data relevant to the first episode were collected and analyzed. Ethical approval was obtained from the medical ethics committee of the Children’s Hospital of Chongqing Medical University.

### 2.2 Data collection

Clinical data from the period preceding the diagnosis of Enterobacteriaceae infection were retrieved from the hospital’s electronic medical record management system and organized using Excel spreadsheets. Patients with recurrent Enterobacteriaceae infections were only included based on their first positive result in this study. The collected clinical information comprised the following: (1) demographics/birth: age, gender, gestational age, birth weight, etc. (2) types of clinical specimens from which CRE and CSE isolates were obtained: blood, sputum, pus, etc. (3) clinical symptom before CRE/CSE isolation: abdominal distension, shock, disseminated intravascular coagulation (DIC), etc. (4) laboratory parameters before CRE/CSE isolation: white blood cell count (WBC), C-reactive protein (CRP), etc. (5) relevant treatments before CRE/CSE isolation: nasogastric tube, surgery, tracheal intubation. (6) pre-existing comorbidities and clinical conditions before CRE/CSE isolation: respiratory failure, hypoproteinemia, congenital heart disease, atrial septal defect, etc. (7) in-hospital outcomes: in-hospital outcomes were classified into five categories according to discharge status: cured, clinically stable discharge, death, treatment withdrawal, and transfer. Cured was defined as cases explicitly documented as “cured” or equivalent terms in the medical record. Clinically stable discharge was defined as patients whose condition improved or stabilized following treatment and who were considered suitable for discharge or outpatient follow-up by the attending physician. Death was defined as death during hospitalization. Treatment withdrawal was defined as discontinuation of inpatient treatment at the family’s request before medical discharge criteria were met, including discharge against medical advice, signed discharge, or withdrawal of life-sustaining treatment. Transfer was defined as transfer to another medical institution for continued treatment.

To ensure data quality and consistency, the following measures were implemented: (1) a standardized data collection form was developed and tested on a random sample of 30 patients prior to formal data collection. (2) regular quality checks were performed throughout the data collection period to identify and correct any errors or omissions. (3) for microbiological data, all isolates underwent species identification and antimicrobial susceptibility testing following standardized protocols with appropriate quality control strains (*Enterobacter aerogenes* ATCC 13048, *Escherichia coli* ATCC 25922, and *Pseudomonas aeruginosa* ATCC 27853), as described in Section 2.6.

### 2.3 Strains

In this study, the term “Enterobacteriaceae” refers specifically to *Escherichia coli, K. pneumoniae*, *Citrobacter freundii*, *Enterobacter cloacae, and Klebsiella oxytoca*. CRE were defined as isolates that were resistant to at least one carbapenem antibiotic (ertapenem, meropenem, or imipenem), according to the Clinical and Laboratory Standards Institute (CLSI) 2022criteria (meropenem and imipenem, ≥4 μg/mL; ertapenem, ≥2 μg/mL). CSE were defined as isolates susceptible to all carbapenems (meropenem, imipenem, and ertapenem) based on CLSI 2022 breakpoints. If multiple isolates were obtained from the same patient, only the first isolate was included in the analysis. All isolates were initially identified using matrix-assisted laser desorption ionization-time-of-flight mass spectrometry (MALDI-TOF MS, bioMérieux, France). *Enterobacter aerogenes* ATCC 13048 was employed as quality- control strains.

### 2.4 Carbapenem resistance gene, extended-spectrum β-lactamase gene, and virulence gene detection

The presence of carbapenem resistance genes (*bla_NDM_*, *bla_KPC_*, *bla_OXA-48_*, *bla_VIM_* and *bla_IMP_*), extended-spectrum β-lactamase (ESBL) genes (*bla_SHV_*, *bla_TEM_* and *bla_CTX-M_*), and virulence genes (*peg-344, rmpA*, *rmpA2*, *iucA* and *iroB*) was detected using polymerase chain reaction (PCR). Primers were synthesized by Sangon Biotech (Shanghai) Co., Ltd. The primer sequences and annealing temperatures for specific genes are provided in **Supplementary Table 1**. All positive PCR products were sequenced by Sangon Biotech (Shanghai, China) and the resulting sequences were analyzed using the BLAST algorithm available from GenBank (www.ncbi.nlm.nih.gov/blast/).

### 2.5 MLST analysis

Multilocus sequence typing (MLST) was performed according on the schemes available on the PubMLST (https://pubmlst.org/) and Institut Pasteur (https://bigsdb.pasteur.fr/) websites. For Escherichia coli, eight housekeeping genes (*dinB, icdA, pabB, polB, putP, trpA, trpB*, and *uidA*)were amplified by PCR, while for *Klebsiella pneumoniae*, seven housekeeping genes (*rpoB, gapA, mdh, pgi, phoE, infB*, and *tonB*) were amplified. Primers were synthesized by Sangon Biotech (Shanghai, China). The primer sequences and annealing temperatures for specific genes are detailed in **Supplementary Table 1**. The PCR products were subjected to bidirectional Sanger sequencing. The resulting sequences were assembled and compared with the MLST database (https://bigsdb.pasteur.fr/index.html) to assign allele numbers and determine sequence types (STs). The phylogenetic tree was constructed using MEGA11 software. For phylogenetic analysis, nucleotide sequences were aligned using ClustalW implemented in MEGA11 software. A phylogenetic tree was constructed using the neighbor-joining (NJ) method with 1,000 bootstrap replicates to assess the robustness of the tree topology.

### 2.6 Antimicrobial susceptibility

Minimum inhibitory concentrations (MICs) of meropenem-avibactam, ceftazidime-avibactam, aztreonam-avibactam, polymyxin B, and tigecycline were determined by broth microdilution method. For meropenem-avibactam (MEM/AVI), aztreonam-avibactam (ATM/AVI), and ceftazidime-avibactam (CAZ/AVI), avibactam was fixed at a concentration of 4 μg/mL, while meropenem, aztreonam, or ceftazidime was added at different concentrations ranging from 0.064 to 64 μg/mL. Susceptibility to other antimicrobial agents was determined using the VITEK 2 automatic system (bioMérieux, France). These antibiotics were selected because they represent current therapeutic options for CRE infections, including novel β-lactam/β-lactamase inhibitor combinations and last-resort agents. *Escherichia coli* ATCC 25922 and *Pseudomonas aeruginosa* ATCC 27853 were used as quality control strains. Antimicrobial susceptibility testing (AST) was performed in triplicate on three separate days. The MIC breakpoints for tigecycline and polymyxin B were interpreted according to the European Committee on Antimicrobial Susceptibility Testing (EUCAST) criteria (https://www.eucast.org), while the others agents were based on the Clinical and Laboratory Standards Institute (CLSI) criteria. MIC results were analyzed using WHONET version 5.6 (http://www.whonet.org/contact.html).

### 2.7 Statistical methods

All univariate and multivariable logistic regression analyses were performed using IBM SPSS Statistics version 25.0, while least absolute shrinkage and selection operator (LASSO) regression was conducted using R software (version 4.4.1). To identify the most important predictors of CRE infection and reduce the risk of overfitting, LASSO regression was employed for variable selection. Variables with a *P* < 0.05 in the univariate analysis were included in the LASSO model. The optimal penalty parameter (λ) was determined using ten-fold cross-validation based on the minimum cross-validated error (lambda.min; λ = 0.03285734). Variables selected by LASSO were subsequently entered into a multivariable logistic regression model to identify independent risk factors for CRE infection. Results were expressed as odds ratios (ORs) with corresponding 95% confidence intervals (CIs). All statistical tests were two-sided, and a *P* < 0.05 was considered statistically significant. The discriminative ability of the final multivariable binary logistic regression model was evaluated using receiver operating characteristic curve analysis. Predicted probabilities were generated from the final model incorporating cesarean section, age, WBC count, and patent ductus arteriosus and were used as the test variable. The area under the curve and its 95% confidence interval were calculated.

### 2.8 Sample size

This study was a retrospective analysis that included all eligible neonatal cases or bacterial strains collected from the two participating hospitals between 2018 and 2022 that met the inclusion criteria. Due to the retrospective nature of the study and the relatively low incidence of CRE infections in neonates, no formal *a priori* sample size calculation was performed. Instead, we included all available cases or strains during the study period to maximize the statistical power and ensure the representativeness of the findings.

### 2.9 Patient consent

This retrospective study was conducted using anonymized patient data, with no involvement of identifiable personal information. The study protocol was reviewed and approved by the institutional Ethics Committee of the Children’s Hospital of Chongqing Medical University, which granted an exemption from informed consent.

## 3 Results

### 3.1 Clinical information of CRE isolates

A total of 139 non-duplicate CRE isolates were collected in this retrospective study. Among these, 113 (81.3%) were carbapenem-resistant *Klebsiella pneumoniae* (CRKP), and 26 (18.7%) were carbapenem-resistant *Escherichia coli* (CREC), all of which were isolated from neonatal patients. The mean age of the patients was 8 days, and 80 (57.6%) were male. The isolates were predominantly obtained from respiratory tract specimens (58.3%, 81/139), followed by blood specimens (24.5%, 34/139), and other sources (17.2%, 24/139). The age distribution and additional clinical characteristics are presented in **Table 1**.

**Table 1:** Clinical characteristics of CRE isolates.

Basic information		Number of cases	(%)
Strain name	CREC	26	18.7
	CRKP	113	81.3
Source	sputum	81	58.3
	blood	34	24.5
	pus	13	9.4
	urine	2	1.4
	drainage fluid	1	0.7
	secretion	5	3.6
	ascites	1	0.7
	catheter	1	0.7
	other	1	0.7
Patients	male	80	57.6
	female	59	42.5
Age(days)	0-10	30	21.6
	11-20	67	48.2
	21-28	42	30.2

### 3.2 Distribution of carbapenemase genes and extended-spectrum β-lactamase genes in CRE isolates

**Figure 1:**
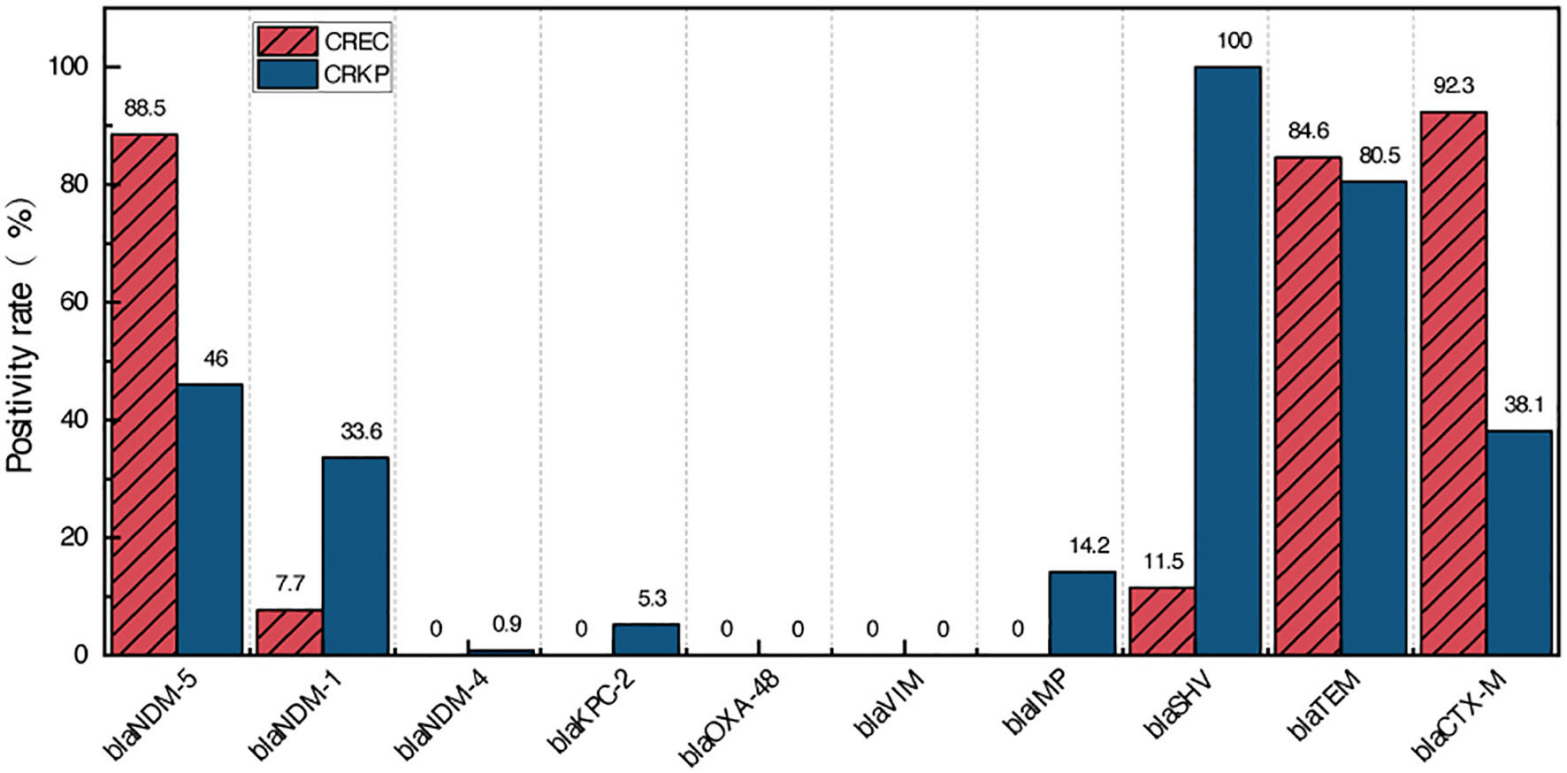
Prevalence of carbapenemase genes and extended-spectrum β-lactamase genes in CRE isolates.

All 139 CRE isolates were subjected to PCR and sequencing to detect ESBL and carbapenemase genes, including *bla_NDM_, bla_KPC,_ bla_OXA-48_, bla_VIM_, bla_IMP,_ bla_SHV_, bla_TEM_*, and *bla_CTX-M_* (**Figure 1**). Carbapenemase genes were detected in 99.3% (138/139) of the CRE isolates. Among the 26 CREC isolates, 96.2% (25/26) harbored *bla_NDM_*, with *bla_NDM-5_* being the predominant variant (88.5%, 23/26), followed by *bla_NDM-1_* (7.7%, 2/26). No CREC isolates were found to carry *bla_KPC_*, *bla_OXA-48_*, *bla_VIM_*, or *bla_IMP_*. Notably, one CREC strain did not harbor any of the targeted carbapenemase genes. For the 113 CRKP isolates, the most prevalent carbapenemase gene was *bla_NDM_* (80.5%, 91/113), with the following distribution: *bla_NDM-5_* (46.0%, 52/113), *bla_NDM-1_* (33.6%, 38/113), and *bla_NDM-4_* (0.9%, 1/113). In addition, *bla_IMP_* and *bla_KPC_* were detected in 14.2% (16/113) and 5.3% (6/113) of CRKP isolates, respectively. No CRKP isolates carried *bla_OXA-48_* or *bla_VIM_*. ESBL genes were widely distributed among both CREC and CRKP isolates. In CRKP isolates, *bla_SHV_, bla_TEM_*, and *bla_CTX-M_* exhibited high detection rates. In contrast, among CREC isolates, only *bla_TEM_* and *bla_CTX-M_* were predominant, while *bla_SHV_* was detected at a low frequency (11.5%, 3/26).

### 3.3 MLST distribution of CRE isolates

**Figure 2:**
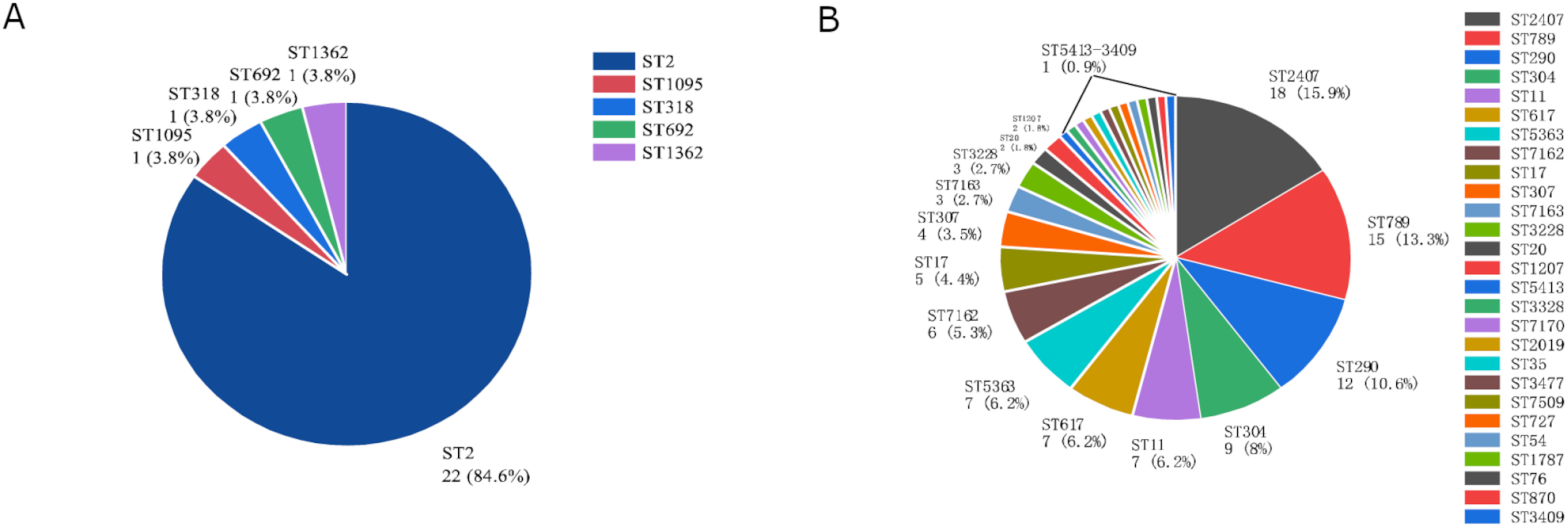
MLST distribution of CRE isolates. **(A)** Sequence types **(STs)** of CREC strains. **(B)** Sequence types (STs) of CRKP strains.

**Figure 3:**
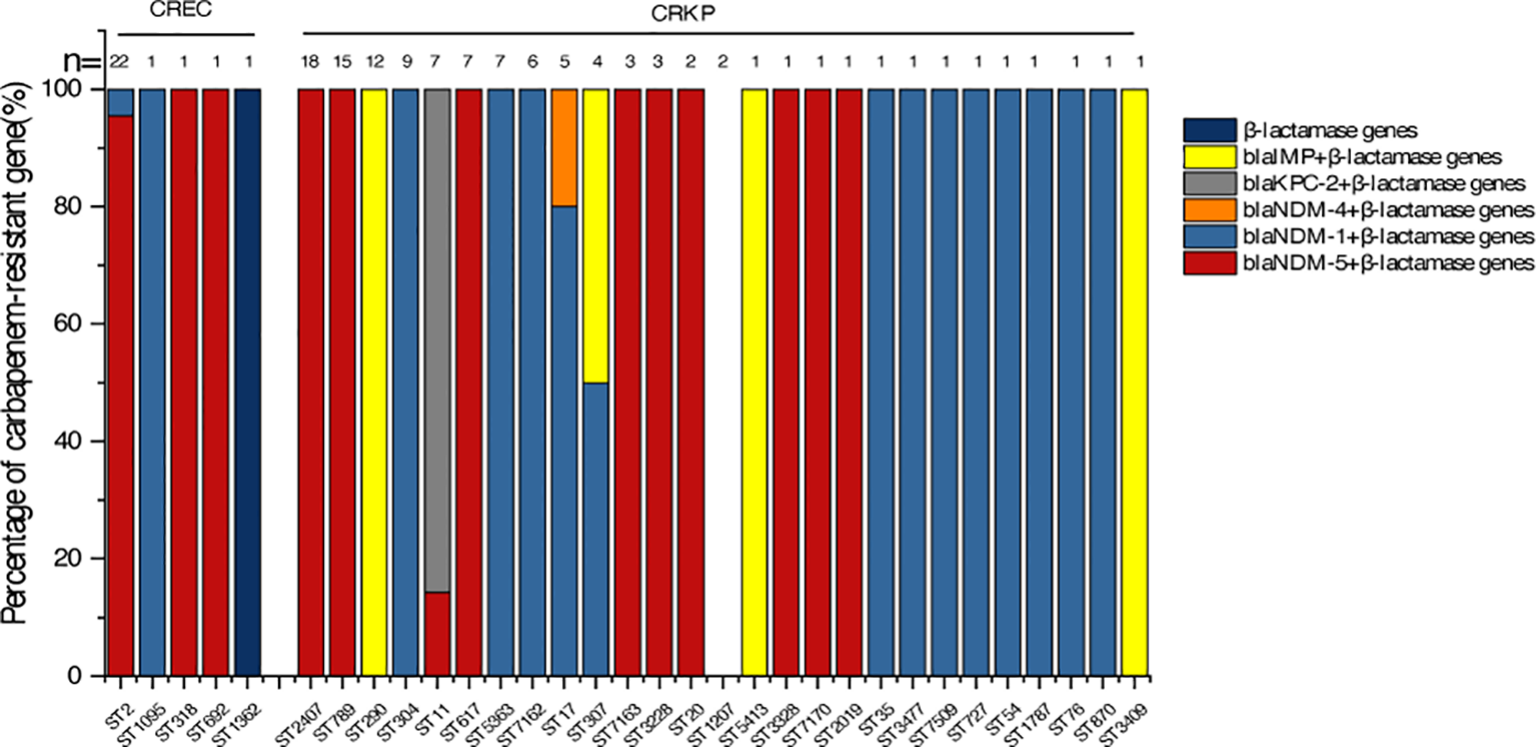
Relationship between sequence types (STs) and resistance genes in CREC and CRKP isolates.

**Figure 4:**
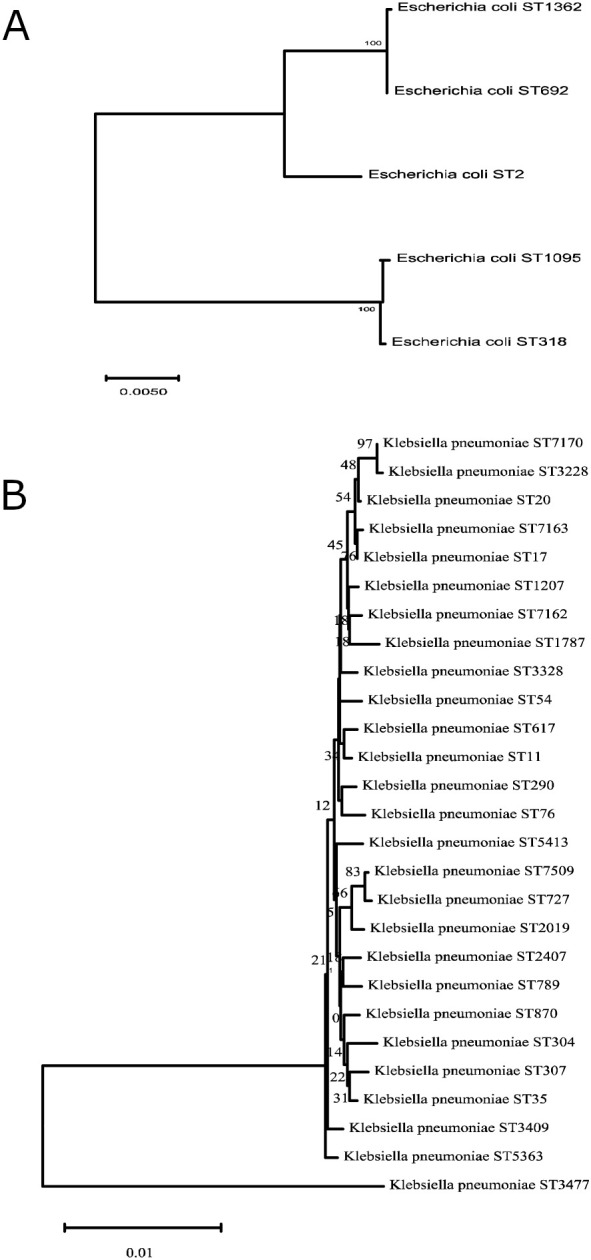
Phylogenetic tree of CRE isolates. **(A)** CREC strains. **(B)** CRKP strains. A phylogenetic tree was constructed using the neighbor-joining (NJ) method with 1,000 bootstrap replicates to assess the robustness of the tree topology.

To investigate the molecular characteristics of the predominant CRE isolates, MLST typing was performed on 26 CREC isolates and 113 CRKP isolates. Among the 26 CREC isolates, MLST analysis identified five different STs. ST2 was the most prevalent, accounting for 84.6% (22/26) of CREC isolates. Other STs, including ST1905, ST318, ST692, and ST1362, were each detected in a single isolate (**Figure 2A**). Among the ST2 CREC isolates, 95.5% (21/22) produced NDM-5 (**Figure 3**). A total of 27 different STs were identified among the 113 CRKP isolates, including four novel STs: ST7162, ST7163, ST7170, and ST7509. ST2407 (15.9%,18/113) was the predominant ST, followed by ST789 (13.3%,15/113), ST290 (10.6%,12/113), ST304 (8.0%,9/113), ST11 (6.2%,7/113), ST617 (6.2%,7/113), ST5363 (6.2%,7/113) (**Figure 2B**). All ST2407 and ST789 CRKP isolates produced NDM-5, while all ST304 CRKP isolates produced NDM-1. In addition, all ST11 CRKP isolates expressed KPC-2, and all ST290 CRKP Isolates produced imipenemase (IMP) (**Figure 3**). Phylogenetic analysis revealed that among CREC isolates, ST1362 exhibited the highest degree of evolutionary divergence (**Figure 4A**). Among CRKP isolates, ST3477 showed a relatively independent evolution and the greatest evolutionary distance, followed by ST1787 (**Figure 4B**).

### 3.4 Antimicrobial susceptibility testing of CRE isolates

The detailed antimicrobial resistance profiles of the 139 CRE isolates are summarized in **Table 2**. All CRE isolates from neonatal patients exhibited high-level resistance (100%) to cephalosporin antibiotics. Similarly, all isolates were resistant to the carbapenems tested, including imipenem (IPM), meropenem (MEM), and ertapenem (ETP). For aztreonam, the resistance rates were 71.7% in CRKP and 84.6% in CREC. While none of the CREC isolates were resistant to aztreonam-avibactam, 3.5% of CRKP isolates were resistant to this combination. Resistance to ceftazidime-avibactam and meropenem-avibactam was observed in 100% of CREC isolates, whereas CRKP isolates exhibited resistance rates of 93.8% and 92.9%, respectively. Regarding aminoglycosides, CRKP isolates exhibited higher resistance rates to gentamicin (20.4%) and amikacin (6.2%) compared with CREC isolates (3.8% and 0%, respectively). For quinolones, CRKP isolates showed lower resistance rates to ciprofloxacin (26.6%) and levofloxacin (10.6%) than CREC isolates (96.2% for both agents). Resistance to sulfamethoxazole/trimethoprim was observed in 42.5% of CRKP isolates and 92.3% of CREC isolates. Notably, none of the isolates in this study were resistant to tigecycline or colistin. In addition, KPC-2-producing strains showed (100%) susceptible to ceftazidime-avibactam.

**Table 2:** Antimicrobial susceptibility of 139 CRE isolate.

Type of antibiotic	Antimicrobials	Total (n=139)				*E. coli* (n=26)				*K. pneumoniae* (n=113)			
		R,n(%)	MIC50	MIC90	MIC range	R,n(%)	MIC50	MIC90	MIC range	R,n(%)	MIC50	MIC90	MIC range
β-lactam	cefuroxime	100	≥64	≥64	≥64	100	≥64	≥64	≥64	100	≥64	≥64	≥64
	ceftriaxone	100	≥64	≥64	≥64-≥128	100	≥64	≥64	≥64	100	≥64	≥64	≥64-≥128
	ceftazidime	100	≥64	≥64	≥64	100	≥64	≥64	≥64	100	≥64	≥64	≥32-≥64
	cefepime	100	≥64	≥64	≥16-≥64	100	≥64	≥64	≥16-≥64	100	≥64	≥64	≥16-≥64
	cefperazone-sulbactam	100	≥64	≥64	≥64	100	≥64	≥64	≥64	100	≥64	≥64	≥64
	piperacillin-tazobactam	100	≥128	≥128	≥64-≥128	100	≥128	≥128	≥64-≥128	100	≥128	≥128	≥64-≥128
	ceftazidime-avibactam	95.0	≥64/4	≥64/4	1/4-≥64/4	100	≥64/4	≥64/4	≥64/4	93.8	≥64/4	≥64/4	1/4-≥64/4
carbapenem	Ertapenem	100	≥8	≥8	2-≥8	100	≥8	≥8	≤0.5-≥8	100	≥8	≥8	2-≥8
	imipenem	100	≥16	≥16	4-≥16	100	≥16	≥16	4-≥16	100	≥16	≥16	4-≥16
	Meropenem	100	≥16	≥16	4-≥16	100	≥16	≥16	≥16	100	≥16	≥16	4-≥16
	Meropenem -avibactam	94.2	≥64/4	≥64/4	0.125/4-≥64/4	100	≥64/4	≥64/4	8/4-≥64/4	92.9	≥64/4	≥64/4	0.125/4-≥64/4
monobactams	aztreonam	74.1	≥64	≥64	≤1-≥64	84.6	≥64	≥64	≤1-≥64	71.7	≥32	≥64	≤1-≥64
	aztreonam-avibactam	2.9	0.125/4	1/4	≤0.064/4-32/4	0	0.5/4	0.5/4	≤0.064/4-8/4	3.5	0.064/4	1/4	≤0.064/4-32/4
aminoglycoside	amikacin	5.0	≤2	≤2	≤2-≥64	0	≤2	≤2	≤2	6.2	≤2	≤2	≤2-≥64
	gentamicin	17.3	≤1	≥16	≤1-≥16	3.8	≤1	≤1	≤1-≥16	20.4.	≤1	≥16	≤1-≥16
fluoroquinolone	ciprofloxacin	38.8	0.5	4	≤0.25-≥8	96.2	≥4	≥4	≤0.25-≥4	26.6	≤0.25	4	≤0.25-≥8
	levofloxacin	27.3	1	8	≤0.25-≥8	96.2	8	8	≤0.25-≥8	10.6	1	1	≤0.25-≥8
sulfonamide	sulfamethoxazole/trimethoprim	51.8	4	≥16	≤0.5-≥16	92.3	≥16	≥16	≤0.5-≥16	42.5	1	16	≤0.5-≥16
colistin	colistin	0	1	1	0.5-2	0	1	1	0.5-≥1	0	1	2	0.5-2
tigecyclines	tigecycline	0	0.5	0.5	0.125-2	0	0.25	0.5	0.25-0.5	0	0.5	0.5	0.125-≥2

### 3.5 Virulence genes in CRKP isolates

**Figure 5:**
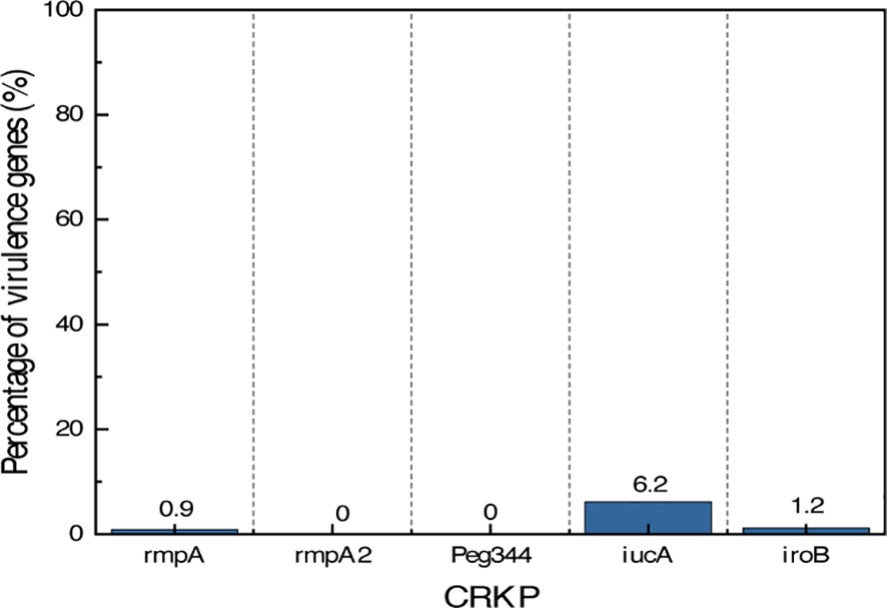
Prevalence of virulence genes in CRKP isolates.

Given the high pathogenicity of *Klebsiella pneumoniae* in neonatal CRE infections and the established association between its virulence genes, (e.g., *rmpA*, *iucA*, and *peg-344*) and hypervirulent phenotypes, this study focused on the detection and analysis of virulence genes in *K. pneumoniae*. Five virulence genes were investigated in 113 CRKP isolates, including capsule synthesis-related genes (*rmpA*, *rmpA2*), the transport-related gene peg-344, and iron uptake-related genes (*iucA*, *iroB*). The detection rates of these virulence genes among the 113 CRKP isolates were as follows: *iucA*(6.2%, 7/113), *rmpA* (0.9%, 1/113), *iroB* (1.8%, 2/113) while *rmpA2* and peg*-344* were not detected in any isolate (**Figure 5**). Among all CRKP isolates, only one strain (belonging to ST17) was found to carry two virulence genes simultaneously (*rmpA* and *iroB*). Notably, all seven *iucA*-positive isolates belonged to ST5363.

### 3.6 Analysis of risk factors for carbapenem-resistant Enterobacteriaceae infection

To facilitate early identification of high-risk neonates and optimize hospital infection control strategies, we conducted a risk factor analysis for CRE infections in neonates. After screening, a total of 245 patients (142 males and 103 females) were included in this study. The CRE group included 124 patients, while the CSE group comprised 121 patients, the baseline characteristics of the two groups are presented in **Table 3**. A total of 68 clinical indicators listed in the baseline characteristics table were included in univariate analysis to assess their association with neonatal CRE isolation. The analysis revealed that 18 factors were significantly associated with CRE infection (*P* ≤ 0.05), including preterm birth, cesarean section, birth weight, gestational age, age, hospital stay duration, surgery, respiratory failure, neonatal respiratory distress syndrome, neonatal asphyxia, patent ductus arteriosus, atrial septal defect, hypoproteinemia, coagulation disorders, hyperbilirubinemia, pulmonary hypertension, symptomatic diarrhea, and white blood cell (WBC) count (**Table 3**). No significant differences were observed between the two groups regarding specimen type, pre-infection clinical symptoms, or in-hospital outcomes.

**Table 3:** Baseline characteristics of CRE and CSE groups.

Variable		CRE (n=124)	CSE (n=121)	Z/X2	P
Demographics & birth	female	51(41.1%)	52(43.0%)	0.086	0.770
	age (days, median [IQR])	1 (0–13)	14 (5–22)	-5.263	0.000<0.05
	birth weight (g, median [IQR])	2315(1700–3070)	2920 (2190-3650)	-4.061	0.000<0.05
	gestational Age (weeks, median [IQR])	35 (32–38)	38 (35–39)	-3.226	0.001<0.05
	hospital stay duration	24 (11–40)	12 (6–22)	-4.160	0.000<0.05
	preterm birth	76(62.8%)	48(40.0%)	12.549	0.000<0.05
	cesarean section	95(76.6%)	65(53.7%)	14.818	0.001<0.05
Specimen type	blood	37(29.8%)	36(29.8%)	0.000	0.988
	sputum	65(52.4%)	68(56.2%)	0.352	0.553
	pus	14(11.3%)	11(9.1%)	0.323	0.570
	urine	1(0.8%)	1(0.8%)	/	1.000
	drainage fluid	1(0.8%)	0(0.0%)	/	1.000
	secretion	5(4.0%)	4(3.3%)	/	1.000
	ascites	1(0.8%)	1(0.8%)	/	1.000
Clinical symptom	dyspnea	67(54.0%)	54(44.6%)	2.167	0.141
	abdominal distension	5(4.0%)	5(4.1%)	0.000	1.000
	shock	20(16.1%)	10(8.3%)	3.525	0.060
	DIC	3(2.4%)	1(0.8%)	/	0.622
	convulsions	7(5.6%)	3(2.5%)	0.863	0.353
Laboratory parameters	WBC (10^9/L, median [IQR])	12.58 (7.98–17.66)	10.31 (7.67–13.45)	-2.056	0.040<0.05
	PLT (10^9/L, median [IQR])	322 (170–438)	313 (178–416)	-0.060	0.953
	HGB (g/L, median [IQR])	131 (114–155)	136 (121–151)	-1.229	0.219
	CRP (mg/L, median [IQR])	8 (8–25)	8 (8–12)	-1.736	0.082
Invasive procedures	nasogastric tube	10(8.1%)	9(7.4%)	0.034	0.855
	surgery	34(27.4%)	20(16.5%)	4.227	0.040<0.05
	tracheal Intubation	28(22.6%)	17(14.0%)	2.973	0.085
Comorbidities and clinical conditions	respiratory Failure	49(39.5%)	25(20.7%)	10.328	0.001<0.05
	neonatal respiratory distress syndrome	27(21.8%)	10(8.3%)	8.718	0.003<0.05
	neonatal pneumonia	99(79.8%)	90(74.4%)	1.035	0.309
	neonatal Asphyxia	21(16.9%)	10(8.3%)	4.166	0.041<0.05
	bronchopulmonary dysplasia	9(7.3%)	9(7.4%)	0.003	0.957
	intracranial hemorrhage	19(15.3%)	17(14.0%)	0.079	0.778
	gastrointestinal hemorrhage	19(15.3%)	14(11.6%)	0.740	0.390
	hemangioma	0(0.0%)	2(1.7%)	/	0.243
	thrombocytopenia	22(17.7%)	13(10.7%)	2.449	0.118
	thrombocytosis	13(10.5%)	6(5.0%)	2.613	0.106
	coagulation disorders	34(27.4%)	11(9.1%)	13.721	0.000<0.05
	acid-base metabolism disorders	39(31.5%)	33(27.3%)	0.515	0.473
	hypoproteinemia	59(47.6%)	37(30.6%)	7.429	0.006<0.05
	hypoglycemia	17(13.7%)	12(9.9%)	0.844	0.358
	hyponatremia	2(1.6%)	6(5.0%)	/	0.168
	hypocalcemia	13(10.5%)	14(11.6%)	0.074	0.786
	hyperlactatemia	38(30.6%)	40(33.1%	0.164	0.685
	hyperkalemia	18(14.5%)	13(10.7%)	0.789	0.375
	hyperbilirubinemia	64(51.6%)	46(38.0%)	4.576	0.032<0.05
	congenital heart disease	2(1.6%)	1(0.8%)	/	1.000
	pulmonary hypertension	31(25.0%)	12(9.9%)	9.627	0.002<0.05
	patent ductus arteriosus	56(45.2%)	19(15.7%)	25.020	0.000<0.05
	atrial septal defect	86(69.4%)	56(46.3%)	13.381	0.000<0.05
	aortic/Tricuspid regurgitation	5(4.0%)	10(8.3%)	1.908	0.167
	premature rupture of membranes	26(21.0%)	18(14.9%)	1.542	0.214
	conjunctivitis	3(2.4%)	4(3.3%)	/	0.720
	dermatitis	5(4.0%)	5(4.1%)	0.000	1.000
	laryngitis	2(1.6%)	2(1.7%)	/	1.000
	gastroesophageal reflux	2(1.6%)	7(5.8%)	/	0.100
	lactose intolerance	2(1.6%)	3(2.5%)	/	0.681
	neonatal necrotizing small bowel colitis	25 (20.2%)	15 (12.4%)	2.703	0.100
	allergic enteritis	0(0.0%)	2(1.7%)	/	0.243
	perianal abscess	3(2.4%)	0(0.0%)	/	0.247
	neonatal acne	2(1.6%)	3(2.5%)	/	0.681
	symptomatic diarrhea	10(8.1%)	26(21.5%)	8.803	0.003<0.05
In-hospital outcomes	Cured	8 (6.5%)	5 (4.1%)	0.656	0.418
	Clinically stable Discharge	75 (60.5%)	82 (67.8%)	1.412	0.235
	Death	2 (1.6%)	0 (0.0%)	/	0.498
	Treatment withdrawal	37 (29.8%)	28 (23.1%)	1.410	0.235
	Transfer	2 (1.6%)	2 (1.7%)	/	1.000

Continuous variables were first tested for normality using the Shapiro-Wilk test. Non-normally distributed data are presented as medians (interquartile ranges) and compared using the Mann-Whitney U test. Categorical variables are expressed as counts and percentages, and differences between groups were assessed using the chi-square test or Fisher’s exact test. The fisher exact test has no X^2^ value and is denoted by/.

**Figure 6:**
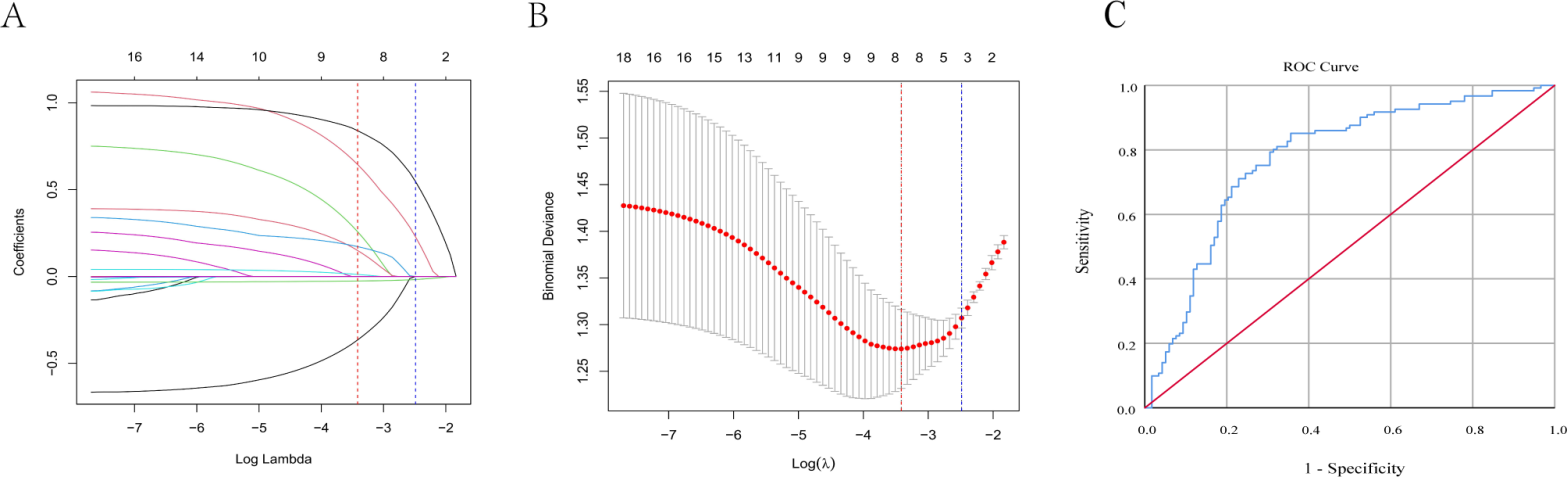
Variable selection using LASSO regression and performance evaluation of the final multivariable binary logistic regression model. **(A)** LASSO coefficient profiles of the candidate variables. Each curve represents the coefficient trajectory of a variable as the regularization parameter (λ) changes. **(B)** Selection of the optimal tuning parameter (λ) using 10-fold cross-validation. The optimal λ value (λ = 0.03285734) was determined based on the minimum mean cross-validated error (lambda.min). **(C)** Receiver operating characteristic (ROC) curve of the final multivariable binary logistic regression model for distinguishing neonatal carbapenem-resistant Enterobacterales (CRE) infection from carbapenem-susceptible Enterobacterales (CSE) infection. The final model incorporated cesarean section, age, white blood cell count, and patent ductus arteriosus, with an AUC of 0.776 (95% CI: 0.716–0.837, P < 0.001).

To reduce the risk of overfitting, LASSO regression was used for variable selection. Excluding hospital stay duration, variables with *P* < 0.05 in the univariate analysis were included in the LASSO model (**Figures 6A, B**). Eight variables were selected by LASSO (cesarean section, surgery, patent ductus arteriosus, atrial septal defect, coagulation disorders, symptomatic diarrhea, age, white blood cell count). These variables were subsequently entered into a multivariable logistic regression to identify independent risk factors for CRE infection. The results showed that cesarean section (OR 3.283, 95% CI 1.682–6.411), age (OR 0.965, 95% CI 0.934–0.998), WBC count (OR 1.049, 95% CI 1.001–1.100), and patent ductus arteriosus (OR 2.758, 95% CI 1.374–5.534) were independent risk factors for CRE infection in neonatal patients (all *P* < 0.05) (**Table 4**).

**Table 4:** Multivariate logistic regression analysis of CRE infection in neonates.

*Factors*	*B*	*SE*	*Wald*	*P*		*OR*	*95%CI*
Cesarean section	1.189	0.341	12.129	0.000<0.05	3.283		1.682~6.411
Age	-0.035	0.017	4.373	0.037<0.05	0.965		0.934~0.998
WBC	0.048	0.024	4.021	0.045<0.05	1.049		1.001~1.100
Surgery	0.703	0.396	3.154	0.076	2.020		0.930~4.389
Patent ductus arteriosus	1.014	0.355	8.148	0.004<0.05	2.758		1.374~5.534
Atrial septal defect	0.463	0.338	1.883	0.170	1.589		0.820~3.080
Coagulation disorders	0.266	0.445	0.357	0.550	1.304		0.546~3.117
Symptomatic diarrhea	-0.628	0.458	1.879	0.170	0.533		0.217~1.310

To assess model performance, ROC curve analysis was performed using the predicted probabilities derived from the final multivariable binary logistic regression model (**Figure 6C**). The final model, which incorporated cesarean section, age, white blood cell count, and patent ductus arteriosus, showed an AUC of 0.776 (95% CI: 0.716–0.837, P < 0.001), indicating acceptable discriminative ability for distinguishing neonatal CRE infection from CSE infection.

## 4 Discussion

Over the past two decades, the resistance rate of Enterobacteriaceae to carbapenem antibiotics has increased dramatically (Hu et al., 2017; Zhang Y. et al., 2018; Zong et al., 2021). The spread of CRE has become a major concern in pediatric infections due to its association with higher morbidity and mortality, as well as limited therapeutic options. The phenotypic and genotypic characteristics of the CRE isolates, along with the risk factors for infection, may be different between neonatal and non-neonatal pediatric patients. Therefore, it is urgent to understand the prevalence of carbapenemase types, the molecular epidemiological features of CRE isolates in neonates, and to identify risk factors for CRE infection in comparison with infections caused by non-carbapenemase-producing strains.

In neonatal patients at our hospital, CRE primarily caused respiratory and urinary tract infections, with CRKP being the most prevalent pathogen. These findings are consistent with prior epidemiological studies (Han et al., 2020; Montagnani et al., 2016; Yen et al., 2023; Yu et al., 2022). Molecular characterization revealed that CRKP isolates predominantly carried NDM-5, NDM-1, and IMP carbapenemase genes, with NDM-5 being the most prevalent among CREC isolates. Additionally, CRKP exhibited significantly greater genetic diversity compared with CREC. CREC isolates were classified into five sequence types (ST2, ST1905, ST318, ST692, and ST1362), whereas CRKP isolates spanned 27 distinct STs. Among CRKP, ST2407 (15.9%) and ST789 (15.1%) were the predominant types, aligning with reports from neonatal populations in other southwestern Chinese provinces (e.g., Sichuan and Guizhou) (Wu et al., 2023; Zhang et al., 2022). The marked predominance of ST2407 in neonatal infections merits further discussion. ST2407 has emerged as a regional clone carrying *bla_NDM-5_* and has shown a tendency to affect pediatric/neonatal settings specifically. When compared with globally recognized high-risk CRKP clones—such as ST11 (KPC-2, predominant in adults worldwide)—ST2407 remains geographically restricted. However, the fact that ST2407 is the dominant clone in a large neonatal cohort suggests that it may have adapted to the neonatal intensive care environment, possibly through enhanced biofilm formation or a unique combination of resistance and colonization factors (Zeng et al., 2024). In contrast, ST11, the dominant clone in Children’s hospitals in eastern China (e.g., Nanjing and Shanghai Children’s Hospitals), mirrored the prevalent ST among Chinese adults (Tao et al., 2022; Yin et al., 2023; Yu et al., 2022). These findings suggest both geographic and age-related differences in CRKP genotypes. Notably, all KPC-2-producing CRKP isolates belonged to ST11, except for one ST301 strain, a pattern consistent with prior studies (Zhang X. et al., 2018). In contrast, NDM-1, NDM-5, and IMP-producing isolates exhibited diverse clonal backgrounds: NDM-5 was primarily associated with ST2407, ST789, ST617, and ST17; IMP was detected in ST290, ST307, ST3409, and ST5413; NDM-1 was distributed across multiple STs (ST304, ST5363, ST7162, ST17, ST1207, ST307, ST35, ST54, ST76, ST727, ST870, ST1787, ST3477, and ST7059). This variability suggests that NDM-1, NDM-5, and IMP genes may be located on highly mobile genetic elements, facilitating their dissemination. Consequently, rigorous screening and continuous genomic surveillance of CRKP isolates are essential for infection control in neonatal patients.

Carbapenem-resistant hypervirulent *Klebsiella pneumoniae* (CR-hvKP) has been increasingly reported in adults in China (Liu et al., 2020, Liu et al., 2023; Zhou et al., 2021). However, to date, few reports have described hvKP infections in neonatal patients. In this study, we used the five genotypic biomarkers proposed by Russo et al (Russo et al., 2018). to accurately identify hvKP. Our findings indicates that hvKP has a low detection rate in neonatal CRKP isolates. Li et al. (2021) reported that none of their neonatal CRKP isolates carried hypervirulence genes (Li et al., 2021). Nevertheless, CR-hvKP warrants attention in neonatal populations, as infected neonates may experience more severe complications and face greater treatment challenges compared with adults.

In the study, we found that carbapenem resistance genes and ESBL genes coexisted in 99.3% of neonatal CRE isolates, further compounding treatment difficulties in neonatal patients. Antimicrobial resistance analysis showed that all CRE isolates were highly resistant to most cephalosporins, β-lactam/β-lactamase inhibitor combinations, monobactams, and carbapenem antibiotics, consistent with previous studies (Wu et al., 2023; Yin et al., 2023). For quinolone antibiotics, CRKP isolates demonstrated moderate resistance, while CREC isolates exhibited high-level resistance. Both CRKP and CREC isolates demonstrated low resistance to amikacin. None of the CRE isolates in this study were resistant to tigecycline or colistin, likely reflecting the limited use of these agents in neonates due to concerns over toxicity and adverse effects. Avibactam (AVI), a non-β-lactam β-lactamase inhibitor, exhibits broad-spectrum activity against serine β-lactamases (including class A, class C, and some class D enzymes). However, it does not inhibit metallo-β-lactamases (MBLs; class B) or protect β-lactams from MBL-mediated hydrolysis (Abboud et al., 2016; Ehmann et al., 2013; Keepers et al., 2014; Lagacé-Wiens et al., 2014; Toussaint and Gallagher, 2015). In contrast, aztreonam (ATM) remains stable against MBL hydrolysis but is susceptible to degradation by most serine β-lactamases, including extended-spectrum β-lactamases (ESBLs), AmpC β-lactamase (AmpC), and KPC enzymes (Sader et al., 2018). Given their complementary mechanisms of action, the combination of ATM and AVI (ATM/AVI) demonstrates potent antimicrobial activity against CRE, including MBL-producing isolates. In the present study, 95.0% of neonatal CRE isolates co-harboredboth MBL-encoding genes and ESBL genes. As anticipated, antimicrobial susceptibility testing showed that all CRE isolates exhibited high resistance to ceftazidime-avibactam and meropenem-avibactam but remained highly susceptible to aztreonam-avibactam, particularly NDM-producing strains. However, clinical efficacy and safety in neonates require further validation in prospective clinical studies.

Risk factors for CRE infection and/or colonization have been widely reported in adults but remain poorly characterized in neonates. Previous studies showed that mechanical ventilation, cesarean section, prior exposure to broad-spectrum β-lactam antibiotics, central venous catheterization, and low birth weight were risk factors for neonatal CRE infection and/or colonization (Darda et al., 2023; Wang et al., 2022; Yen et al., 2023). In this study, neonates in the CRE group exhibited a higher prevalence of underlying conditions, including respiratory, cardiovascular, and gastrointestinal diseases. These patients were also more likely to have undergone mechanical ventilation, central venous catheterization, surgical procedures, and prior exposure to broad-spectrum β-lactam antibiotics, which placed them at a greater risk of CRE infection. Symptomatic diarrhea may contribute to disruption of the gut microbiota, potentially facilitating CRE colonization and subsequent infection. Our findings align with previous studies that identified cesarean section and younger age as independent risk factors for CRE infection in neonates. Importantly, this study is among the first to identify WBC count as an independent risk factor for CRE infection in this population. This may reflect a paucity of neonatal-specific data linking immune markers to multidrug-resistant infections. Previous studies have indicated a close relationship between WBC count and the occurrence of infections caused by multidrug-resistant organisms (Yin et al., 2024). Additionally, patent ductus arteriosus was identified as another independent risk factor for CRE infection in neonates. This may be explained by the hemodynamic consequences of patent ductus arteriosus, including increased pulmonary blood flow, which can lead to prolonged pulmonary congestion, impaired respiratory defense mechanisms, and heightened susceptibility to respiratory infections, including those caused by CRE.

## 5 Study limitations

Several limitations of this study should be acknowledged. First, this study focused exclusively on Enterobacteriaceae and therefore did not provide a comprehensive overview of the full pathogen spectrum associated with neonatal infections. In future research, we plan to expand the scope to include a broader range of pathogens to achieve a more complete understanding of the neonatal infection landscape. Second, the retrospective design of this study limits our ability to establish definitive causal relationships. Certain variables—particularly length of hospital stay and specific comorbidities—may have complex bidirectional associations with CRE infection. Prolonged hospitalization may increase the risk of CRE acquisition due to extended environmental exposure and invasive procedures, whereas CRE infection itself may lead to clinical deterioration and prolonged hospitalization. Similarly, some clinical conditions (e.g., neonatal respiratory distress syndrome [NRDS], pneumonia) may predispose neonates to CRE infection, while others may be exacerbated by the infection. To minimize this bias, we only included comorbidities diagnosed before CRE isolation and calculated hospital stay duration as a post-infection outcome rather than a predictor. Nevertheless, residual confounding may still exist, and prospective cohort studies are warranted to better establish temporal relationships. Third, the clinical management of CRE-positive neonates is an important aspect of patient care. Although we collected data on in-hospital outcomes and length of hospital stay, we did not systematically evaluate specific treatment strategies. Future prospective studies should focus on treatment approaches and their impact on clinical outcomes in neonates with CRE infection. Finally, our data primarily reflect the epidemiological and antimicrobial resistance characteristics of CRE in the neonatal wards of two children’s hospitals during the 2018–2022 period. Therefore, the current resistance profile should be further validated and updated through future studies with larger sample sizes, multicenter collaborations, and continuous surveillance. Although the final model showed acceptable discriminative ability, it was developed and evaluated in the same retrospective cohort without external validation. Therefore, its clinical applicability should be interpreted cautiously, and further prospective multicenter studies are required to validate its performance in independent neonatal populations.

## 6 Conclusion

In conclusion, the production of NDM is the main mechanism of carbapenem resistance in neonatal CRE isolates. *Klebsiella pneumoniae* ST2407 is a highly prevalent clone in this population and warrants continuous genomic surveillance. ATM/AVI demonstrated potent *in vitro* activity against neonatal CRE isolates. Importantly, cesarean section, patent ductus arteriosus, and younger age were identified as significant risk factors for CRE infection, highlighting the need for targeted screening and enhanced antimicrobial stewardship in high-risk neonates.

## Data Availability

The datasets presented in this study can be found in online repositories. The names of the repository/repositories and accession number(s) can be found in the article/**Supplementary Material**.

## Footnotes

1 Centers for Disease Control and Prevention (CDC). *Antibiotic resistance threats in the United States*. 2022. Available at: https://www.cdc.gov/drugresistance/biggest-threats.html (accessed 3.9.24).

2 Tacconelli E, Magrini N, Carmeli Y, et al. *Global Priority List of Antibiotic-Resistant Bacteria to Guide Research, Discovery, and Development of New Antibiotics*. 2017. Available at: https://www.aidsdatahub.org/sites/default/files/resource/who-global-priority-list-antibiotic-resistant-bacteria.pdf.
