# Supplementary material for: Molecular epidemiology and clinical profiles of carbapenem-resistant Enterobacterales in neonates from two large children’s hospitals in southwestern China
Source: Front Cell Infect Microbiol. 2026 May 21;16:1735917. doi: 10.3389/fcimb.2026.1735917 (PMC13233677; doi:10.3389/fcimb.2026.1735917)
Supplement: Supplementary file 1 [file Table1.docx]

Supplementary Material

# Table S1

| Gene name | | Sequence | Annealing Temperature（℃） |
| --- | --- | --- | --- |
| Carbapenemase Genes and Extended-Spectrum β-Lactamase Genes | | |  |
| KPC | F | ATGTCACTGTATCGCCGTCTA | 56 |
|  | R | TTACTGCCCGTTGACGCCCAA |  |
| NDM | F | ATGGAATTGCCCAATATTATG CAC | 56 |
|  | R | TCA GCG CAG CTT GTC GGC |  |
| OXA-48 | F | GAATGCCTGCGGTAGCAAAG | 56 |
|  | R | GGGCGATCAAGCTATTGGGA |  |
| VIM | F | GTTTGGTCGCATATCGCAAC | 56 |
|  | R | ATGAGCAGCACCAGGATAGA |  |
| IMP | F | CATGGTTTGGTTGTTCTTGT | 56 |
|  | R | ATAATTTAGCGGACTTTGGC |  |
| TEM | F | AGTATTCAACATTTTCGTGT | 56 |
|  | R | TAATCAGTGAGGCACCTATCTC |  |
| SHV | F | ATGCGTTATATTCGCCTGTG | 56 |
|  | R | TTAGCGTTGCCAGTGCTC |  |
| CTX-M | F | TTAGGAAGTGTGCCGCTGCA | 56 |
|  | R | CGATATCGTTGGTGGTRCCAT |  |
| Virulence Genes | | |  |
| rmpA | F | ACTGGGCTACCTCTGCTTCA | 56 |
|  | R | CTTGCATGAGCCATCTTTCA |  |
| rmpA2 | F | CTTTATGTGCAATAAGGATGTT | 56 |
|  | R | CCTCCTGGAGAGTAAGCATT |  |
| iroB | F | ATCTCATCATCTACCCTCCGCTC | 62 |
|  | R | GGTTCGCCGTCGTTTTCAA |  |
| iucA | F | AATCAATGGCTATTCCCGCTG | 65 |
|  | R | CGCTTCACTTCTTTCACTGACAGG |  |
| peg-344 | F | CTTGAAACTATCCCTCCCTCCAGTC | 55 |
|  | R | CCAGCGAAAGAATAACCCC |  |
| MLST-CREC | | |  |
| dinB | F | GTTTTCCCAGTCACGACGTTGTATGAGAGGTGAGCAATGCGTA | 56 |
|  | R | TTGTGAGCGGATAACAATTTCCGTAGCCCCATCGCTTCCAG |  |
| icdA | F | GTTTTCCCAGTCACGACGTTGTAATTCGCTTCCCGGAACATTG | 56 |
|  | R | TTGTGAGCGGATAACAATTTCATGATCGCGTCACCAAAYTC |  |
| pabB | F | GTTTTCCCAGTCACGACGTTGTAAATCCAATATGACCCGCGAG | 56 |
|  | R | TTGTGAGCGGATAACAATTTCGGTTCCAGTTCGTCGATAAT |  |
| polB | F | GTTTTCCCAGTCACGACGTTGTAGGCGGCTATGTGATGGATTC | 56 |
|  | R | TTGTGAGCGGATAACAATTTCGGTTGGCATCAGAAAACGGC |  |
| putB | F | GTTTTCCCAGTCACGACGTTGTACTGTTTAACCCGTGGATTGC | 58 |
|  | R | TTGTGAGCGGATAACAATTTCGCATCGGCCTCGGCAAAGCG |  |
| trpA | F | GTTTTCCCAGTCACGACGTTGTAGCTACGAATCTCTGTTTGCC | 58 |
|  | R | TTGTGAGCGGATAACAATTTCGCTTTCATCGGTTGTACAAA |  |
| trpB | F | GTTTTCCCAGTCACGACGTTGTACACTATATGCTGGGCACCGC | 56 |
|  | R | TTGTGAGCGGATAACAATTTCCCTCGTGCTTTCAAAATATC |  |
| uidA | F | GTTTTCCCAGTCACGACGTTGTACATTACGGCAAAGTGTGGGTCAAT | 56 |
|  | R | TTGTGAGCGGATAACAATTTCCCATCAGCACGTTATCGAATCCTT |  |
| MLST-CRKP | | |  |
| rpoB | F | GGCGAAATGGCWGAGAACCA | 56 |
|  | R | GAGTCTTCGAAGTTGTAACC |  |
| gapA | F | TGAAATATGACTCCACTCACGG | 56 |
|  | R | CTTCAGAAGCGGCTTTGATGGCTT |  |
| mdh | F | CCCAACTCGCTTCAGGTTCAG | 60 |
|  | R | CCGTTTTTCCCCAGCAGCAG |  |
| pgi | F | GAGAAAAACCTGCCTGTACTGCTGGC | 60 |
|  | R | CGCGCCACGCTTTATAGCGGTTAAT |  |
| phoE | F | ACCTACCGCAACACCGACTTCTTCGG | 56 |
|  | R | GAGTTTGTTATCGCTTTTCAGC |  |
| infB | F | CTCGCTGCTGGACTATATTCG | 56 |
|  | R | CGCTTTCAGCTCAAGAACTTC |  |
| tonB | F | CTTTATACCTCGGTACATCAGGTT | 60 |
|  | R | ATTCGCCGGCTGRGCRGAGAG |  |
