# Supplementary material for: Molecular epidemiology and clinical profiles of carbapenem-resistant Enterobacterales in neonates from two large children’s hospitals in southwestern China
Source: Front Cell Infect Microbiol. 2026 May 21;16:1735917. doi: 10.3389/fcimb.2026.1735917 (PMC13233677; doi:10.3389/fcimb.2026.1735917)
Supplement: Supplementary file 2 [file Table2.docx]

**The following list presents the novel sequence types (STs) identified in this study, including accessible query URLs and corresponding gene sequences.**

**Website:** [Search or browse profiles](https://bigsdb.pasteur.fr/cgi-bin/bigsdb/bigsdb.pl?db=pubmlst_klebsiella_seqdef&page=query)

**Novel gene subtype sequences：**

**rpoB 387：**

ACCCACTACGGTCGCGTATGTCCGATCGAAACGCCTGAAGGTCCGAACATCGGTCTGATT

AACTCCCTGTCCGTGTACGCGCAGACCAACGAATATGGCTTCCTTGAGACGCCGTATCGT

AAAGTGACCAACGGTGTGGTTACTGACGAAATTCACTACCTGTCTGCTATCGAAGAAGGC

AACTACGTTATCGCTCAGGCGAACTCCAACCTGGATGAAAACGGCCACTTCGTAGAAGAT

CTGGTTACCTGCCGTAGCAAAGGCGAATCCAGCTTGTTCAGCCGCGACCAGGTTGACTAC

ATGGACGTATCCACCCAGCAGGTGGTATCCGTCGGTGCGTCCTTGATCCCGTTCCTGGAA

CACGATGACGCCAACCGTGCATTGATGGGTGCGAACATGCAACGTCAGGCGGTTCCGACT

CTGCGCGCTGATAAGCCGCTGGTTGGTACCGGTATGGAACGTGCTGTTGCCGTTGACTCC

GGTGTTACTGCCGTGGCTAAA

**tonB 972**：

ATGGTGGCGCCGGCCGATCTTGAGCCGCCTCCGGCGGCGCAGCCTGTCGTGGAGCCCGTT

GTTGAACCCGAACCTGAGCCGGAGCCAGAGGTAGCGCCTGAACCGCCGAAAGAGGCGCCG

GTGGTGATCCATAAACCGGAACCTAAGCCGAAGCCCAAACCTAAACCCAAGCCTAAGCCG

GAGAAAAAGGTTGAACAGCCGAAGCGGGAAGTGAAGCCGGCAGCAGAGCCGCGTCCGGCC

TCGCCGTTTGAAAACAACAATACGGCGCCGGCGCGTACAGCGCCAAGTACCTCGACCGCA

GTGGCTAAACCCACCGTTACTGCTCCGAGCGGCCCGCGGGCGATCAGCCGCGTTCAGCCG

TCCTATCCGCCGCGCGCTCAGGCGCTGCGCATTGAAGGGACGGTACGGGTGAAG

**The novel sequence types：**

| ST | gapA | infB | mdh | pgi | phoE | rpoB | tonB |
| --- | --- | --- | --- | --- | --- | --- | --- |
| 7162 | 4 | 1 | 1 | 1 | 12 | 387 | 4 |
| 7163 | 2 | 1 | 1 | 1 | 4 | 4 | 972 |
| 7170 | 4 | 3 | 1 | 37 | 3 | 27 | 4 |
| 7509 | 2 | 9 | 2 | 1 | 7 | 1 | 5 |
